# Automated coronary artery tracking in contrast-enhanced whole-heart coronary magnetic resonance angiography at 3.0T

**DOI:** 10.1186/1532-429X-15-S1-W30

**Published:** 2013-01-30

**Authors:** D Dey, A Schuhbaeck, Q Yang, Z Fan, G Germano, S Achenbach, D Li, P Slomka

**Affiliations:** 1 Biomedical Imaging Research Institute, Cedars-Sinai Medical Center, Los Angeles, CA, USA; 2 Department of Internal Medicine II, University of Erlangen, Erlangen, Bavaria, Germany; 3 Department of Radiology, Xuanwu Hospital, Beijing, China

## Background

Contrast-enhanced whole heart coronary Magnetic Resonance Angiography (MRA) at 3.0 Tesla (T) allows noninvasive detection of obstructive stenoses. Automated vessel segmentation and tracking of centerlines is important for quantitative measurement of stenosis, but remains challenging for coronary MRA. We aimed to develop and validate automated coronary artery segmentation from contrast-enhanced whole-heart coronary Magnetic Resonance Angiography at 3.0T.

## Methods

Fifteen patients underwent contrast-enhanced whole-heart coronary MRA using electrocardiograph-triggered, navigator-gated gradient-echo sequences, at 3.0T, with voxel size of 0.625x0.625x0.9 mm. Automated coronary vessel tracking (AVT) was performed by an algorithm which simultaneously searches for the vascular centerlines and radius, by maximizing a vessel likelihood cost function derived from the directional radial gradients computed from coronary MRA, from a manually placed proximal to a distal end voxel. The algorithm incorporates expected vessel tapering and performs Dijkstra-based search for the optimal path. Forty-five coronary arteries (Left Main and Left Anterior Descending Artery [LM-LAD], Left Circumflex [LCX], Right Coronary Artery [RCA]) were analyzed by the algorithm. An expert reader manually marked the centerlines for all the arteries for comparison. Algorithm performance was evaluated by the Euclidian distance from the expert reader in mm over all arterial centerline points, and also assessed visually by the expert reader on a 3-point grading scale (1-poor, 2-good, 3-excellent).

## Results

The average and maximum 3D distance between the expert and AVT was 0.7 ± 0.4 mm and 1.7 ± 1.5 mm, respectively. The mean visual grade was 2 ± 1 in the LM-LAD, 3 ± 1 in the LCX, and 3 ± 1 in the RCA, and 2.5 ± 0.8 overall (see example Figure). AVT was visually judged to be successful (score >=2) in 99/105 (94%) proximal and mid coronary artery segments, and 41/45 (91%) of distal segments; algorithm failure was primarily related to presence of motion artifacts in the corresponding arterial segment.

**Figure 1:**
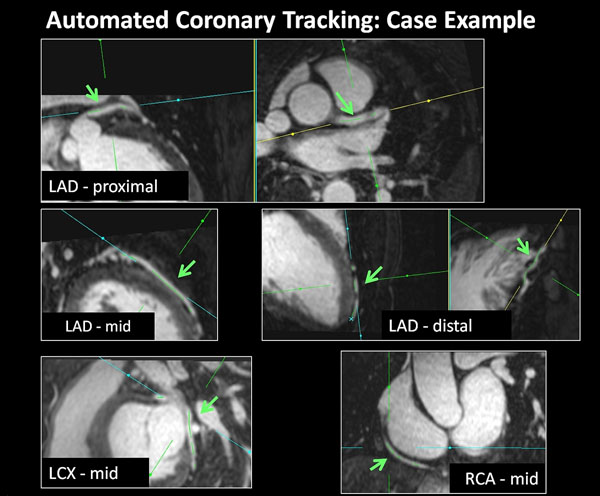


## Conclusions

We developed an automated coronary vessel tracking algorithm which shows promising results for coronary MRA at 3.0T.

